# Supplementary material for: ntStat: k-mer characterization using occurrence statistics in raw sequencing data
Source: PLoS Comput Biol. 2026 Apr 1;22(4):e1014158. doi: 10.1371/journal.pcbi.1014158 (PMC13056179; doi:10.1371/journal.pcbi.1014158)
Supplement: S1 Text — Fig A. Wall clock time of ntStat’s filter command with different arguments vs. the number of threads. The dataset consisted of reads simulated from the C. elegans reference genome and contained 2,135,806,380 k-mers (k = 30). Fig B. Wall clock time of hackgap compared to ntStat for different k-mer sizes and minimum count thresholds (cmin2 and cmin3). Spaced seed patterns “1110111100011100011110111” and “111011110001110011100011110111” are used for s25 and s30, respectively, and k25 and k30 represent k-mers with no spaced seed masking applied. Table A. List of statistics summarized using the histogram model. “Err” and “Peak” refer to the distributions selected for the erroneous and genomic peaks, respectively. The number of k-mers with count i are shown as hi, and c refers to the maximum k-mer count available in the histogram. Each component is parameterized by w and θ, and the final model is represented by f(x). Table B. Datasets used for benchmarking ntStat’s counting module’s performance. Table C. Specifications of the simulated datasets shown in Fig 4. ‘Copy SNV rate’ refers to the -s and -d parameters set when creating the second haplotype using pIRS. ‘Number of robust k-mers’ and ‘number of heterozygous k-mers’ are the total number of k-mer present in at least one and exactly one of the haplotypes, respectively. Percentages of robust and heterozygous k-mers are relative to the total and robust k-mers, respectively. ‘Robust coverage’ is calculated as the number of robust k-mers divided by the total number of k-mers present in the initial assembly. The script we used for obtaining these ground truths is available on ntStat’s GitHub repository. Table D. GenomeScope’s output for the simulated datasets (S3 Table). Table E. Data accession numbers, number of iterations until convergence, and final model error for the histogram analysis experiments on real data. (DOCX) [file pcbi.1014158.s001.docx]

**Supplementary information for “ntStat: k-mer characterization using occurrence statistics in raw sequencing data”**

Parham Kazemi^1,2^, Lauren Coombe^1^, René L. Warren^1^, and Inanc Birol^1,3^

1 BC Cancer Research Institute, Vancouver, Canada

2 Bioinformatics Graduate Program, Faculty of Science, University of British Columbia, Canada

3 Department of Medical Genetics, University of British Columbia, Vancouver, Canada

# Supplementary Figures


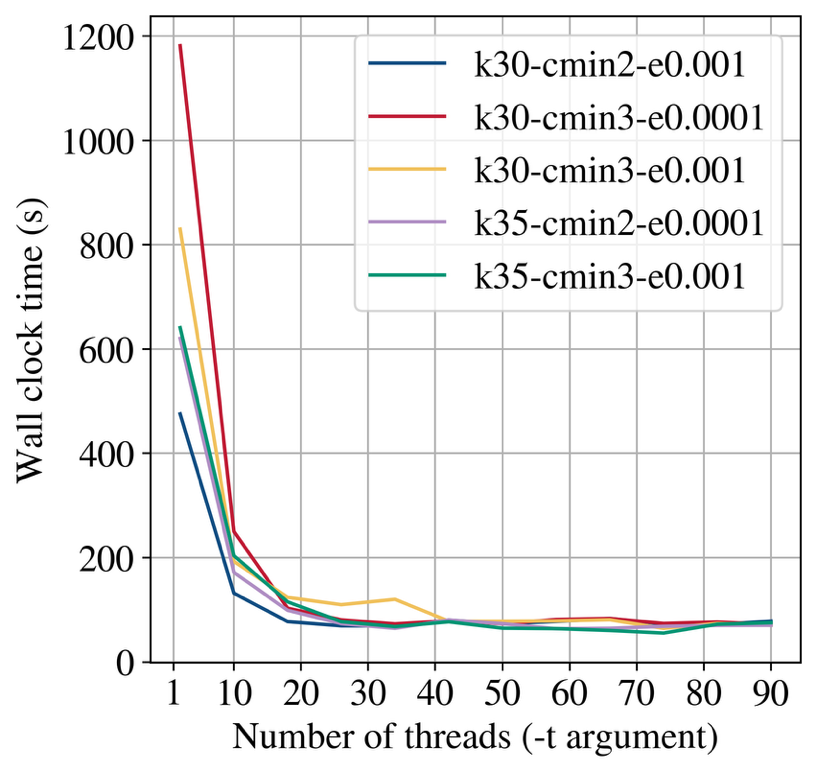


**Fig A.** Wall clock time of ntStat’s filter command with different arguments vs. the number of threads. The dataset consisted of reads simulated from the *C. elegans* reference genome and contained 2,135,806,380 k-mers (k=30).


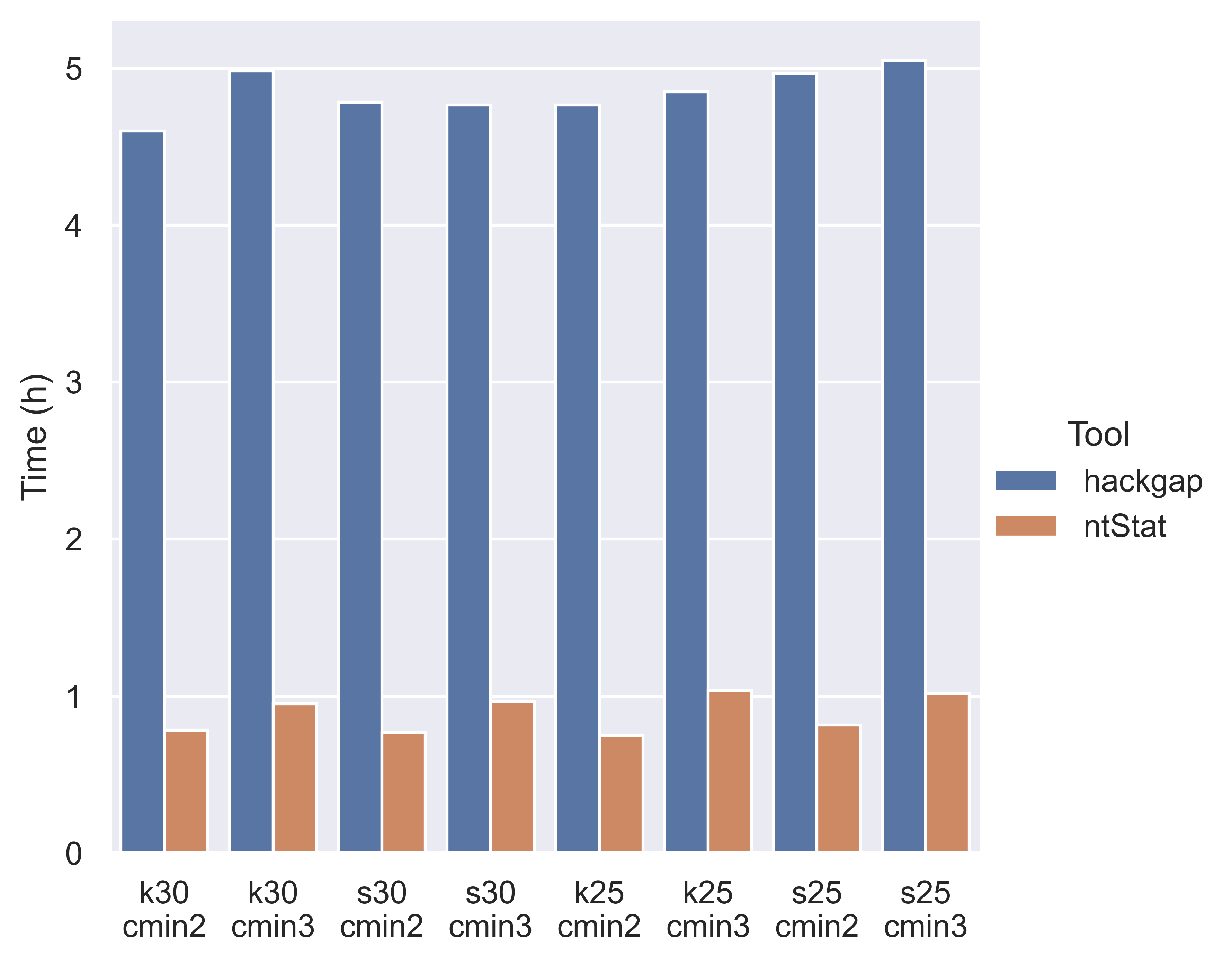


**Fig B.** Wall clock time of hackgap compared to ntStat for different k-mer sizes and minimum count thresholds (cmin2 and cmin3). Spaced seed patterns “1110111100011100011110111” and “111011110001110011100011110111” are used for s25 and s30, respectively, and k25 and k30 represent k-mers with no spaced seed masking applied.

# Supplementary Tables

| **Table A.** List of statistics summarized using the histogram model. “Err” and “Peak” refer to the distributions selected for the erroneous and genomic peaks, respectively. The number of *k*-mers with count *i* are shown as *h_i_*, and *c* refers to the maximum *k*-mer count available in the histogram. Each component is parameterized by $w$ and $\theta$, and the final model is represented by $f\left( x \right)$. | | |
| --- | --- | --- |
| **Characteristic** | **Definition** | **Notation and formula** |
| Dataset size | Estimated by the total number of *k*-mers | $D=\sum_{i} ih_{i}$ |
| Robust *k*-mer rate | Rate of non-erroneous *k*-mers in model | $R= 1-w_{err}c^{-1}\sum_{i} \text{Err}\left( i;\theta_{err} \right)f\left( i \right)^{-1}$ |
| Heterozygosity | Rate of heterozygous *k*-mers in model | $H={c^{-1}w}_{het}\cdot\sum_{i} \text{Peak}\left( i;\theta_{het} \right)f\left( i \right)^{-1}$ |
| Coverage | Mean homozygous *k*-mer coverage | $C$, mean of the homozygous component |
| Genome size | Estimated genome size considering only non-erroneous *k*-mers | $G=RDC^{-1}$ |

**Table B.** Datasets used for benchmarking ntStat’s counting module’s performance.

| **Dataset** | **Accession** | ***k*-mer length** | **Total number of *k*-mers** | **Number of distinct *k*-mers** |
| --- | --- | --- | --- | --- |
| *C. elegans* N2 strain | DRR008444 | 25 | 5,884,298,973 | 222,430,527 |
| *H. sapiens* | SRR11321732 | 25 | 135,611,220,597 | 14,085,447,007 |
|  |  | 64 | 134,535,578,263 | 28,424,193,141 |

**Table C.** Specifications of the simulated datasets shown in Fig. 4. ‘Copy SNV rate’ refers to the -s and -d parameters set when creating the second haplotype using pIRS. ‘Number of robust *k*-mers’ and ‘number of heterozygous *k*-mers’ are the total number of *k*-mer present in at least one and exactly one of the haplotypes, respectively. Percentages of robust and heterozygous *k*-mers are relative to the total and robust *k*-mers, respectively. ‘Robust coverage’ is calculated as the number of robust *k*-mers divided by the total number of *k*-mers present in the initial assembly. The script we used for obtaining these ground truths is available on ntStat’s GitHub repository.

| **Dataset** | **(a)** | **(b)** | **(c)** | | **(d)** | **(e)** | **(f)** |
| --- | --- | --- | --- | --- | --- | --- | --- |
| **GenBank assembly** | *H. sapiens* T2T-CHM13v1.1  (GCA_009914755.3) | | *D. melanogaster*  (GCA_000001215.4) | | | *C. elegans* N2  (GCA_000002985.3) | |
| **Assembly size (bp)** | 3,054,832,041 | | 137,567,484 | | | 100,272,607 | |
| **Copy SNV rate** | 0.1% | 1% | 1% | 1% | | 0.1% | 0.1% |
| **Read simulator** | NanoSim | NanoSim | NanoSim | pIRS | | pIRS | pIRS |
| **Simulator parameters** | -n 5000000 | -n 5000000 | -n 1000000 | --diploid  -x 40 -e 0.001 | | --diploid  -x 50 -e0.001 | --diploid -x 50  -e 0.01 |
| **Dataset size (bp)** | 164,381,284,679 | 164,428,072,145 | 32,883,654,190 | 5,502,615,600 | | 5,013,688,000 | 5,013,688,000 |
| ***K-*mer size** | 30 | 30 | 30 | 35 | | 35 | 25 |
| **Number of *k*-mers in dataset** | 164,091,911,108 | 164,091,911,108 | 32,825,779,470 | 3,631,726,296 | | 3,309,065,100 | 3,810,438,600 |
| **Number of robust *k*-mers** | 134,560,146,583  (82.00%) | 131,425,391,361  (80.09%) | 26,628,412,675  (81.12%) | 3,302,450,522  (90.93%) | | 3,005,781,424  (90.83%) | 2,807,305,806  (73.67%) |
| **Predicted robust rate** | 78.40% | 79.05% | 79.32% | 90.08% | | 90.21% | 71.96% |
| **Robust coverage** | 44.1x | 43.3x | 193.8x | 24.0x | | 30x | 28.0x |
| **Predicted coverage** | 43.7x | 43.8x | 194.3x | 24.7x | | 30.2x | 28.2x |
| **Predicted genome size** | 2.9Gbp | 3.0Gbp | 134.0Mbp | 132.2Mbp | | 98.7Mbp | 97.1Mbp |
| **Number of heterozygous *k*-mers** | 6,804,087,236  (5.05%) | 47,108,199,087  (35.84%) | 11,184,884,056  (34.07%) | 1,553,051,065  (47.02%) | | 192,408,736  (6.40%) | 127,658,986  (4.55%) |
| **Predicted heterozygosity** | 5.01% | 36.50% | 36.99% | 48.50% | | 7.19% | 4.84% |
| **Number of iterations** | 252 | 204 | 235 | 211 | | 59 | 54 |
| **Model error** | 4e-6 | 4e-6 | 3e-6 | 7e-6 | | 1.8e-5 | 1.2e-5 |

| **Table D.** GenomeScope’s output for the simulated datasets (S3 Table). | |
| --- | --- |
| (a)  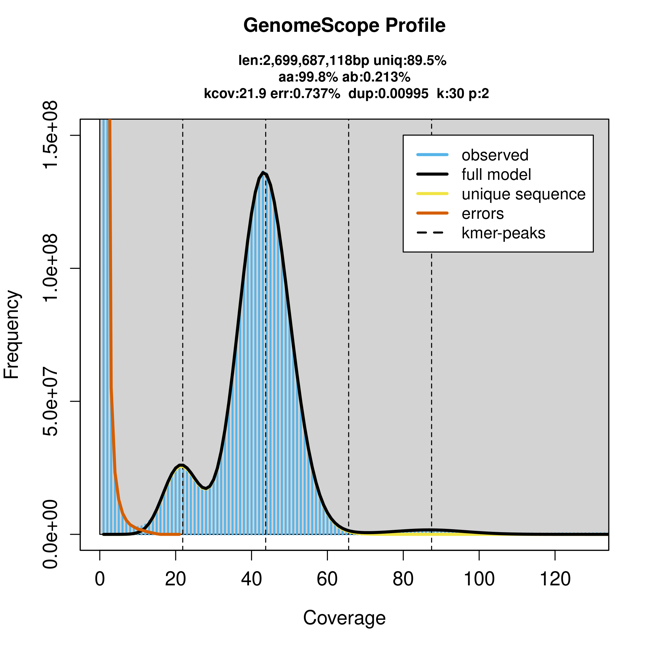  k = 30  property min max  Homozygous (aa) 99.7801% 99.7934%  Heterozygous (ab) 0.206633% 0.21993%  Genome Haploid Length 2,698,126,848 bp 2,699,687,118 bp  Genome Repeat Length 284,562,963 bp 284,727,519 bp  Genome Unique Length 2,413,563,885 bp 2,414,959,599 bp  Model Fit 92.1328% 99.3021%  Read Error Rate 0.73674% 0.73674% | (b)  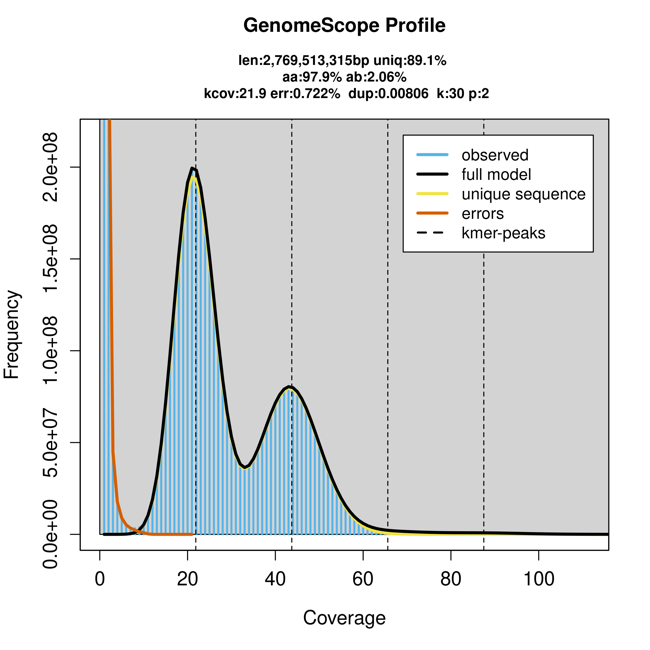  k = 30  property min max  Homozygous (aa) 97.9381% 97.9484%  Heterozygous (ab) 2.05164% 2.06195%  Genome Haploid Length 2,766,947,447 bp 2,769,513,315 bp  Genome Repeat Length 300,597,191 bp 300,875,943 bp  Genome Unique Length 2,466,350,257 bp 2,468,637,372 bp  Model Fit 92.7545% 98.9862%  Read Error Rate 0.721632% 0.721632% |
| (c) 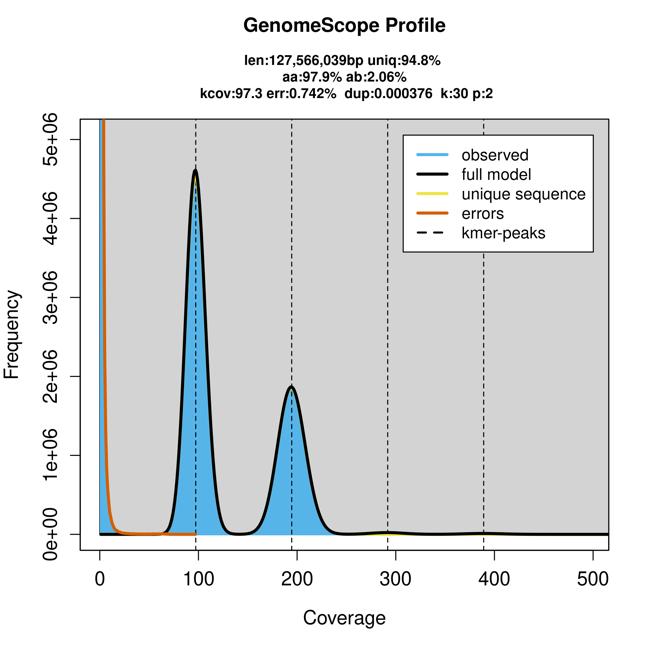  k = 30  property min max  Homozygous (aa) 97.94% 97.9463%  Heterozygous (ab) 2.05373% 2.06004%  Genome Haploid Length 127,536,013 bp 127,566,039 bp  Genome Repeat Length 6,589,734 bp 6,591,285 bp  Genome Unique Length 120,946,280 bp 120,974,754 bp  Model Fit 96.4266% 98.1907%  Read Error Rate 0.741961% 0.741961% | (d)  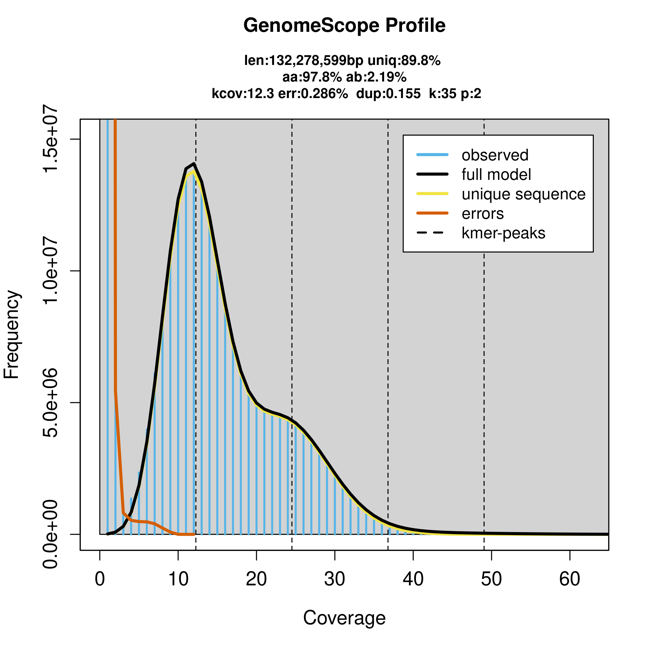  k = 35  property min max  Homozygous (aa) 97.7883% 97.8378%  Heterozygous (ab) 2.1622% 2.21165%  Genome Haploid Length 131,963,582 bp 132,278,599 bp  Genome Repeat Length 13,438,686 bp 13,470,766 bp  Genome Unique Length 118,524,896 bp 118,807,833 bp  Model Fit 91.5246% 98.7005%  Read Error Rate 0.286078% 0.286078% |
| (e)  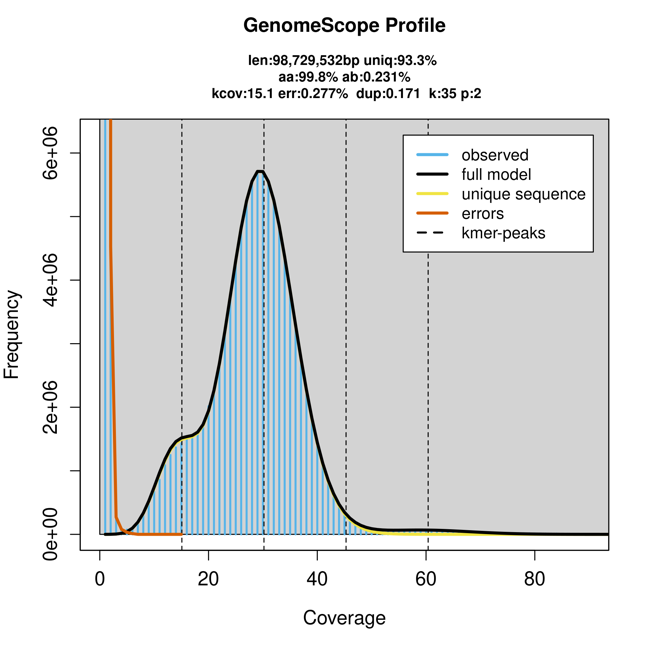  k = 35  property min max  Homozygous (aa) 99.766% 99.7711%  Heterozygous (ab) 0.228873% 0.233982%  Genome Haploid Length 98,678,465 bp 98,729,532 bp  Genome Repeat Length 6,625,957 bp 6,629,386 bp  Genome Unique Length 92,052,508 bp 92,100,146 bp  Model Fit 95.2585% 98.8958%  Read Error Rate 0.277067% 0.277067% | (f)  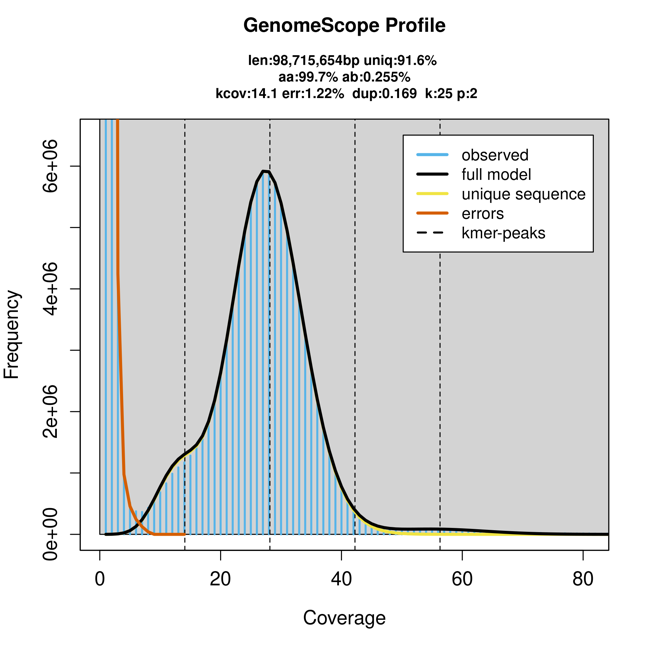  k = 25  property min max  Homozygous (aa) 99.7388% 99.7509%  Heterozygous (ab) 0.249109% 0.261225%  Genome Haploid Length 98,651,211 bp 98,715,654 bp  Genome Repeat Length 8,273,391 bp 8,278,795 bp  Genome Unique Length 90,377,820 bp 90,436,859 bp  Model Fit 94.0392% 98.8462%  Read Error Rate 1.22001% 1.22001% |

| **Table E.** Data accession numbers, number of iterations until convergence, and final model error for the histogram analysis experiments on real data. | | | |
| --- | --- | --- | --- |
| Platform | Accession | Num. iterations | Model error |
| ONT | giab_lsk114_2022.12 | 210 | 8e-6 |
| PacBio | SRX5327410 | 225 | 6e-6 |
| Illumina | SRR11321732 | 206 | 7e-6 |
