# Supplementary material for: ntStat: k-mer characterization using occurrence statistics in raw sequencing data
Source: PLoS Comput Biol. 2026 Apr 1;22(4):e1014158. doi: 10.1371/journal.pcbi.1014158 (PMC13056179; doi:10.1371/journal.pcbi.1014158)
Supplement: Attachment — Submitted filename: ntstat-r2r.pdf [file pcbi.1014158.s002.pdf]

Reviewer #1

This manuscript developed a new software called ntStat for counting k-mers from sequencing data. ntStat has two modules: the k-mer counting module and the histogram analysis module.

The k-mer counting module use bloom filters (BF) and counting bloom filters (CBF) for k-mer counting. It is important to understand the basic ideas of BF and CBF to understand how ntStat works. The basic idea of a bloom filter is that that it uses multiple hash functions for each k-mer, with each hash value corresponds to a bit in the output hash table. If one hash value of the a new k-mer is not set in the hash table, it means that this k-mer has not been counted before. For counting BF, we store the counts of hash values, instead of bool values, which means the count is incremented for each input hash value. The minimum value of the counts over all hash values of a k-mer is used as the count of the input k-mer. This means CBF can overestimate the k-mer count if there is a hash collision.

Due to the usage of bloom filters, ntStat can run faster with less memory, with the tradeoff of accuracy loss. In the results, section 3.3 and 3.4, authors have demonstrated that ntStat are either faster than SOTA methods or use less memeory. The novel features of ntStat are (1) it can filter k-mers (without sorting) whose count numbers are in a predefined range c_min and c_max; (2) it can calculate the k-mer depth information, e.g. how many reads contain a given k-mer. The k-mer depth information can be used to derive the frequency-inverse document frequency (TF-IDF), which are useful for genome assembly tools.

The histogram analysis module is more vaguely described. It seems that the ntStat tries to fit a 3-component mixture model to the k-mer count density plot, with the components representing errors, heterozygous and homozygous. However, the background biological problem is not clearly described and the model specifications are missing, as well as the inference algorithm. In section 3.5-3.6, authors try to illustrate that ntStat can relibly estimate haplotypes given the proportion of error, heterozygous and homozygous components from sequencing data with different configurations.

I think this is decent software paper if authors can address the following major concerns:

1. add a more detailed introduction to BF and CBF with toy examples in the methods

Thank you for pointing this out. We have added a more detailed description of BFs and CBFs to the "Count and depth extraction" section and Fig. 1a in Design and Implementation.

2. add a mathematical formulation of the histgram analysis. what is the research question? what is the mathematical model to address the research question, e.g. the parametric forms of the components? How the inference algorithm works, what is the objective function?

We updated the Histogram analysis module section with more details and a formula defining the mixture model.

3. i do not get the connection between the k-mer counting module and the histogram analysis module. the input to the histogram analysis module are generated by ntCard, which I assume is a published software. i thought the input are generated by the k-mer counting module.

Thank you for identifying this point of confusion. While the counting module is designed for explicit indexing, querying, and retrieval of specific k-mers (which requires constructing large Bloom filters), the histogram analysis module is often used for rapid, global quality control (e.g., genome size or heterozygosity estimation) where the counts of individual k-mers is not needed. We utilize ntCard as the input generator for the histogram module because it employs a streaming algorithm to estimate count distributions, allowing us to generate the required histogram significantly faster and with a smaller memory footprint than the counting module. We have added this explanation to the Histogram analysis section.

4. it was argued that TF-IDF is important, but I did not see authors using it in the applications of the results section. it will be nice to add an application that use this information.

We appreciate the reviewer highlighting the need to connect the theoretical importance of TF-IDF to a practical application in the Results section. Fundamentally, TF-IDF weighs term frequency against document frequency; in our context, the count-to-depth ratio serves as a genomic analogue, where the *k*-mer count represents the term frequency (TF) and the depth (number of reads) represents the document frequency (DF). The application of this metric is demonstrated in the K-mer characterization section (Section 3.4) and Figure 6e, f. We have clarified the wording in the manuscript to emphasize how count to depth ratio can be used to distinguish between different types of repeats.

5. clearly describe the contributions of ntStat. If the histogram analysis part is already known method, then the contribution of ntStat is a re-implementation or extension of GenomeScope.

See our response to point #6 below.

6. can GenomeScope and ntStat be used to analyse the same data? if yes, please add a comparion of the results on the two software on the same data.

The core contributions of ntStat go beyond re-implementing GenomeScope by integrating efficient, k-mer counting with a platform-flexible statistical model. The key value lies in dynamic histogram modeling using evolutionary algorithms to handle high-error long-read data, providing precise k-mer-level characteristics rather than just global genomic metrics. We confirm that GenomeScope and ntStat can analyze the same data, and our direct comparison of results is provided in Supplementary Tables S3 and S4; we trust that the issue of the supplementary materials being unavailable to the reviewer has been resolved in this revision. We have added more content to clarify the distinct problems ntStat and GenomeScope are designed to solve.

7. Supplmentary are not available

We apologize for the issue regarding the Supplementary Information accessibility. We will double-check the files uploaded during the resubmission process.

Minor comments:

1. Fig. 1, the workflow of the couning process is not obvious, try to add more text in the legend and improve the figure, e.g. explain "global" and "depth"

2. section 3.2, first sentence, add ref to ntCard

3. section 3.2, last paragraph, "ntStat reports .... and classifying k-mers". could you elaborate what is "classifying k-mers"?

4. Fig. 2b, add the absolute memory usage corresponding to 0.96 and 1.

5. section 3.3, last paragraph, "....where the area under the count .... for that range is larger", "for that range is larger" => "larger range"

6. section 3.5, first paragraph, "Furthermore, we counted .... and copy as robust", split the sentence into shorter ones and explain what is "copy as robust"

7. section 3.5, paragraph start with "Next, to show ...", "we find that ntStat estimate the rate of robust k-mers ......", what is "robust k-mers"?

8. section 3.6, first paragraph, "p(component = c | x)... the density of the component", F_c(x) is not density, should be "probability". Also, it seems to be more reasonable to define F_c(x) = w_c * f_c(x), where w_c is the prior weight of component c and f_c(x) is the likelihood of x given component c.

We appreciate the reviewer's minor points, which have all been addressed in the new version of the manuscript.

Reviewer #2

The paper introduces a new k-mer counting approach with attention to memory efficiency. The same team already has previous work (ntCard) on the same subject. Here, novel metrics such as frequency–inverse document frequency and histogram fitting are presented.

Main remarks:

The method appears incremental compared to established tools (e.g., KMC, Genoscope), which perform better in their specialized tasks. Reported speed gains may not justify tradeoffs, and reduced disk usage is less compelling given current storage availability (especially on machines equipped with, e.g., many threads as presented in the benchmark).

- I think it is nice to have an indexed output. However how do authors feel about forcing a specific output format and data structure with respect to adoption? For instance, many modern k-mer indexing methods emphasize cache-efficient designs.

We appreciate the reviewer's concern regarding limited adoption caused by using a specific output data structure. We emphasize that the Counting Bloom Filter (CBF) format is utilized not to restrict usage, but as a design choice to achieve balance between query speed, file size, and memory efficiency. To maximize adoption and utility, the tool includes utilities to export the k-mer count and depth information to the standard TSV format.

- Similarly, the emphasis on C++ with Python bindings being "seamless" is more a benefit for the authors' development workflow than for external users in my opinion. The tool has many dependencies and is not especially easy to install.

We appreciate the reviewer's feedback on the user-friendliness of the binding design and the installation complexity. The modular implementation primarily benefits users when integrating into existing Python-based bioinformatics pipelines, as ntStat's API is public. We acknowledge that managing dependencies can be challenging for any tool built manually from source; therefore, to ensure robust and easy installation for the community, we have made ntStat available on Bioconda, and we have updated the readme file on the GitHub repository accordingly.

- Results reported on 48 CPUs are unclear (CPUs vs cores vs threads), could we see scalability curves instead?

We have generated performance curves plotting runtime vs. thread count; these results are now included as a Supplementary Figure S1.

- The need to specify Bloom filter size or error parameters is not user-friendly; many users will not know how to provide these. Can the authors justify their design here?

We understand the reviewer's concern. However, for most common tasks, we have carefully tuned and validated default parameters to provide a robust balance of accuracy and efficiency, allowing standard users to run ntStat without requiring any manual configuration of these settings. Through the error rate parameter (-e), advanced users have the option to set the Bloom filter sizes necessary for their application.

- The manuscript focuses on genomics, and the fitting models are supposed to be adjustable, but the paper does not demonstrate utility in RNA-seq or metagenomics, which could also limit adoption.

We agree with the reviewer that demonstrating the utility of ntStat in other fields, particularly transcriptomics and metagenomics, would broaden its application. While our current evaluation focused strictly on genomic data, the core functionality, namely, the platform-flexible histogram modeling and the extraction of k-mer depth, is theoretically applicable to both fields. The underlying hashing scheme, ntHash2, already supports the RNA alphabet (A, C, G, U), meaning no change to the core counting functionality is required to process RNA-seq data. As noted in the Future Directions section, statistical interpretation of results for RNA-seq or metagenomics data would be different, but ntStat would still generate proper output for the data types.

- The tool is claimed to be useful for long reads, but sequencing error rates are improving for these technologies. To that regard, could you specify ntStat's actual supported range of k values? (I'd like to clarify if large k are supported)

We appreciate the reviewer's observation that long-read sequencing error rates are improving, and large k-mer sizes remain crucial for accurate analysis. We confirm that since ntHash2's theoretical k-mer size limit is 2,042,040bp, ntStat practically supports an unlimited range of k values. We have added a sentence to the manuscript's Design and Implementation section to explicitly state this.

- Key comparisons to GenomeScope are hidden in the supplement. From the text, GenomeScope appears to provide more comprehensive biological insights (heterozygosity, ploidy). The specific added value of ntStat remains unclear. If ntStat is to underpin error-correction methods (as suggested in the paragraph before the conclusion), runtime for recovering erroneous k-mers should be addressed.

We have added more content to clarify the distinct problems ntStat and GenomeScope are designed to solve. Specifically, ntStat is designed to provide *k*-mer-level statistics, such as the probability of an individual *k*-mer containing a sequencing error, whereas GenomScope provides global statistics like the genome's heterozygosity rate.

Minor

- succinct probabilistic Bloom filters : there's no Bloom filter that is not probabilistic. "Succinct" is used twice in the text but there is no precise mention of the actual data structure used to represent the Bloom filter/CBF.

- in 3.2 "unlike previous studies in the literature" add citations

Thank you for mentioning these points, which we have addressed in our revised manuscript.

Reviewer #3

This paper presents ntStat, a software toolkit for characterizing raw sequencing data through k-mer statistics. Its contribution is twofold. First, it provides a k-mer counting module that uses Bloom filters to achieve a balance of speed, low memory usage, and minimal disk footprint compared to existing tools. An innovation of this module is its ability to concurrently track not

only the total frequency of each k-mer but also its "depth", defined as the number of distinct sequencing reads in which it appears. Second, the paper introduces a histogram analysis module that uses evolutionary algorithms to model the distribution of k-mer counts. This approach adapts to data from various sequencing platforms, including high-error long-read technologies, to provide de novo estimates of genomic characteristics such as heterozygosity, sequencing quality, and k-mer coverage.

The paper is well-written and clear, however, the presented results make it somewhat difficult to fully grasp the specific problems that ntStat is designed to solve.

Concerning the histogram analysis, an in-depth comparison between the capabilities and performance of GenomeScope2 and those of ntStat would be beneficial for the manuscript's readability.

We have added more content to clarify the distinct problems ntStat and GenomeScope are designed to solve. ntStat is designed to provide k-mer-level statistics, such as the probability of an individual k-mer containing a sequencing error, whereas GenomScope provides global statistics like the genome's heterozygosity rate.

A similar concern appears regarding the k-mer counting implementation. The implementation uses ideas very similar to BFCounter, so a summary of the key differences between the methods seems necessary. Other efficient k-mer counting tools exist, for instance, Gerbil, which is very efficient for large k (Gerbil: a fast and memory-efficient k-mer counter with GPU-support), or hackgap (Fast Gapped k-mer Counting with Subdivided Multi-Way Bucketed Cuckoo Hash Tables), which follows a similar in-memory approach but with Cuckoo filters. Additionally, the widely used and efficient, though unpublished, tool FASTK should be considered. As a result, the current benchmark is not truly representative of the state of the art. Furthermore, it is somewhat unclear in which situations ntStat actually shines.

We appreciate the reviewer's feedback. We have added hackgap to our comparative analysis, since ntStat and hackgap are the only tools that support spaced seeds. We have also updated the text to explicitly differentiate ntStat from BFCounter by highlighting the thresholding capabilities, depth extraction, and spaced seed support.

Finally, although the notion of k-mer depth is interesting, a discussion regarding the utility of this metric and its differences from classic k-mer abundance seems necessary. Moreover, using a Bloom filter to avoid counting duplicate k-mers within a given read seems computationally expensive and can introduce false positives, as two distinct k-mers could be mapped to the same hash value. A hash table would likely be more efficient and would avoid this unnecessary supplementary bias.

We thank the reviewer for these insightful points. The discussion regarding k-mer depth is expanded to highlight its utility as a metric distinct from classic k-mer abundance. Specifically, depth is crucial for characterizing repetitive elements: k-mers with a high count-to-depth ratio identify local/tandem repeats (occurring multiple times within a single read), a distinction not possible with standard counting.

Regarding the data structure used to track k-mers within a read, we clarify that our original read-specific Bloom filter was dimensioned to be large enough to fit all k-mers in a single read with effectively zero false positives, thereby acting as a hash table. We intentionally favored this implementation over a hash table to avoid the memory overhead associated with explicitly storing keys or pointers (O(N) memory complexity). A standard hash table requires dynamic allocation and significantly more space per entry to handle collisions, whereas our approach uses a fixed-size bit array with a minimal, predictable footprint. Furthermore, because the hash values are already computed for the global counting structures, reusing these pre-calculated hashes to set bits in a local array is more efficient and architecturally compatible than managing a separate hash table.
