# Supplementary material for: ntStat: k-mer characterization using occurrence statistics in raw sequencing data
Source: PLoS Comput Biol. 2026 Apr 1;22(4):e1014158. doi: 10.1371/journal.pcbi.1014158 (PMC13056179; doi:10.1371/journal.pcbi.1014158)
Supplement: Attachment — Submitted filename: R2R.pdf [file pcbi.1014158.s003.pdf]

Reviewer #1

The authors have addressed most of my concerns. After reading the responses and review comments from other reviewers, I find the histogram analysis part meant for applications in genome assembly field, which I have no experience.

The conclusion and discussion are deleted in the revised manuscript. I think it is necessary to keep the discussion/conclusion such that general readers can understand what are the typical applications of this software and what are the difference compared with other software like GenomeScope.

The paper can be published after putting back the discussions/conclusions.

We thank Reviewer 1 for their time and for the constructive feedback provided during the review process. The content we previously had in the discussions is now available in the final subsection of the Results (Section 3.5).

Reviewer #2

The authors have adequately addressed my comments regarding the data structure and the modularity of the tool. They have also added benchmark curves, which improves the evaluation of the method. In addition, they provided more details about the k-mer size and clarified the difference with GenomeScope.

Note: the sentence "NtStat is designed to model the underlying probability of individual k-mers belonging to error, heterozygous or homozygous components" might be better placed in Section 2.2, particularly in relation to another reviewer's comment noting that the paper does not clearly explain the motivation behind the histogram.

We would like to thank Reviewer 2 for their dedicated time and for the helpful suggestions. We agree that the sentence does belong in Section 2.2, and we moved it as suggested.

Reviewer #3

I have re-reviewed the revised version of the manuscript describing ntStat, a toolkit for characterizing raw sequencing data through k-mer statistics, combining an efficient k-mer counting strategy that tracks both k-mer frequency and the "depth" (number of distinct reads containing the k-mer), and a histogram analysis module based on evolutionary algorithms to infer

de novo genomic and sequencing characteristics across a range of sequencing technologies, including error-prone long-read data.

The authors have addressed my previous concerns in a thorough and constructive manner. The revised manuscript now more clearly articulates the distinct scope and aims of ntStat compared to GenomeScope, notably by emphasizing ntStat's ability to provide k-mer-level statistics (e.g., error probability of individual k-mers) rather than only global genome-wide estimates. The manuscript also better differentiates the k-mer counting component from related approaches such as BFCounter by explicitly highlighting ntStat's thresholding capabilities, depth extraction, and spaced seed support. Importantly, the benchmarking section has been strengthened by the inclusion of hackgap, which is particularly relevant given the shared support for spaced seeds, improving the representativeness of the comparison.

In addition, the discussion of k-mer depth has been expanded and is now substantially more convincing, especially regarding its utility for characterizing repetitive elements via the count-to-depth ratio. The authors have also clarified the rationale behind the read-level Bloom filter design, including its sizing to make false positives effectively negligible in this context and the practical benefits of fixed-size memory use and reuse of precomputed hashes.

Overall, I am satisfied with the current state of the manuscript and believe that the revisions have improved clarity, positioning with respect to existing tools, and the motivation for the proposed metrics and design choices. I thank the authors for their diligent work in addressing the review comments.

Sincerely,

We thank Reviewer 3 for their careful reading of the work and for providing constructive feedback.
